# Inflammatory Indices and Preterm Delivery: A New Horizon in Obstetric Risk Assessment

**DOI:** 10.3390/diagnostics15101188

**Published:** 2025-05-08

**Authors:** Samet Kırat

**Affiliations:** Department of Gynecology and Obstetrics, Faculty of Medicine, Kafkas University, Kars 36000, Turkey; sametkirat1989@hotmail.com

**Keywords:** inflammation, lymphocytes, monocytes, neutrophils, preterm delivery

## Abstract

**Objective:** Preterm delivery is a leading cause of neonatal morbidity and mortality globally, with inflammation playing a crucial role in its pathophysiology. This study aimed to evaluate the predictive value of systemic inflammatory response indices in identifying pregnant women at risk of preterm delivery. **Methods:** This retrospective study analyzed data from 1128 pregnant women admitted to a tertiary care hospital between 2020 and 2025. Patients were classified into two groups: preterm delivery (*n* = 528) and term delivery (*n* = 600). Demographic characteristics, obstetric history, neonatal outcomes, and inflammatory indices were compared. **Results:** The preterm delivery group showed a significantly higher systemic inflammatory response index (SIRI) (*p* < 0.001), systemic immune-inflammation index (SII) (*p* < 0.001), neutrophil/lymphocyte ratio (NLR) (*p* < 0.001), and monocyte/lymphocyte ratio (MLR) (*p* < 0.001) than the term delivery group, while platelet/lymphocyte ratio (PLR) levels were significantly lower (*p* = 0.002). Inflammatory indices were higher in early preterm delivery cases (*p* < 0.001) than in middle and late preterm cases. Multivariate logistic regression identified the SIRI (*p* = 0.015) and NLR (*p* < 0.001) as independent predictors of preterm delivery, while the PLR showed an inverse association (*p* = 0.002). Higher inflammatory indices correlated with lower 1st and 5th minute APGAR scores (*p* < 0.001) and increased neonatal intensive care unit (NICU) admission rates (*p* < 0.001). NICU stay was prolonged in neonates born to mothers with elevated SIRI and NLR levels (*p* < 0.001). **Conclusions:** Integrating these inflammatory indices into obstetric risk assessment may enhance early detection and intervention strategies, potentially improving maternal and neonatal prognosis.

## 1. Introduction

Delivery occurring prior to the 37th week of gestation is classified as preterm and remains a significant determinant of adverse neonatal outcomes, including increased risks of morbidity and mortality [1]. Millions of babies are born prematurely every year worldwide, increasing the need for neonatal intensive care units (NICUs) and creating a huge burden on both health systems and families [2]. Although the underlying causes of preterm delivery have not been fully elucidated, recent studies have suggested that inflammation is one of the most important mechanisms [3].

A chronic maternal inflammatory response during pregnancy may lead to an increase in systemic cytokine levels, which may increase uterine contractility and trigger preterm delivery [4]. In particular, levels of inflammatory markers such as interleukin-6 (IL-6), tumor necrosis factor-alpha (TNF-α), C-reactive protein (CRP), and matrix metalloproteinase-9 (MMP-9) have been shown to be significantly higher in women with preterm delivery [5]. In this context, assessing inflammation at the systemic level may help in the early identification of pregnant women at risk of preterm delivery and allow the development of preventive interventions [6].

The systemic inflammatory response index (SIRI) is a biomarker used to assess the severity of inflammation and is calculated based on the peripheral blood cell count [7]. Hematologic parameters such as the systemic immune-inflammation index (SII), neutrophil/lymphocyte ratio (NLR), platelet/lymphocyte ratio (PLR), and monocyte/lymphocyte ratio (MLR) are considered important indicators of inflammatory processes and have been associated with preterm delivery [1]. Recent studies have suggested that these indices can be used to predict preterm delivery and contribute to the development of early intervention strategies [8].

The aim of this study was to evaluate the prognostic value of systemic inflammatory response indices in the early diagnosis of preterm delivery. In particular, the effectiveness of hematologic inflammatory indices such as the SIRI, SII, NLR, PLR, and MLR in predicting preterm delivery will be examined. In this direction, we aimed to determine the differences in the inflammatory response between pregnant women with and without preterm delivery. The main hypothesis of this study was that high inflammatory indices may increase the risk of preterm delivery and that these parameters can contribute to early intervention strategies by integrating them into clinical practice.

## 2. Materials and Methods

### 2.1. Patients and Data Collection

This study, designed as a retrospective, descriptive, and cross-sectional analysis, was conducted on 1128 pregnant women admitted to the Obstetrics and Gynecology Clinic of a tertiary university hospital between January 2020 and January 2025. Singleton pregnancies between the ages of 18 and 55 years with complete blood count data for both the first trimester and at the time of presentation were included in this study. Pregnant women under the age of 18, women with chronic diseases (diabetes mellitus, hypertension, autoimmune disease, etc.), active urinary and/or genital tract infections, pregnancies conceived with assisted reproductive techniques, intrauterine excitus cases, and multiple pregnancies were excluded. Demographic and obstetric characteristics such as age, gravida, parity, miscarriage, smoking, employment status, educational level, and socioeconomic status were evaluated. In addition, the gestational week at the time of admission, APGAR scores at 1 and 5 min, history of NICU admission, and duration of admission were examined. Complete blood count parameters obtained in the first trimester and during admission were analyzed.

The study group consisted of preterm delivery patients (*n* = 528), and the control group consisted of term delivery patients (*n* = 600). The study and control groups were compared, and patients with preterm delivery were divided into three subgroups according to their gestational age: early preterm (<32 + 0 weeks), middle preterm (32 + 0 − 33 + 6 weeks), and late preterm (34 + 0 − 36 + 6 weeks). The relationships between these subgroups were evaluated, and comparisons were made according to the parameters determined.

### 2.2. Inflammatory Indices

#### 2.2.1. Systemic Inflammatory Response Index (SIRI)

The SIRI, calculated as the ratio of the product of the number of monocytes and neutrophils times the number of lymphocytes, is a biomarker assessing inflammatory status. A high SIRI is indicative of strong inflammation and poor immune response. It was first described by Qi et al. in 2016 to predict survival in pancreatic cancer and has been used as a prognostic factor in various diseases [9].

#### 2.2.2. Systemic Immune-Inflammation Index (SII)

It is calculated as the product of the number of neutrophils and platelets times the number of lymphocytes. It is a marker representing both inflammatory and immune responses. High SII levels indicate the severity of inflammation and have been associated with poor prognosis in conditions such as cancer, cardiovascular disease, and infection [10].

### 2.3. Neutrophil/Lymphocyte Ratio (NLR)

It is calculated as the number of neutrophils divided by the number of lymphocytes. Given that neutrophils are the first line of defense against infections, the NLR is considered a reliable parameter for monitoring and assessing the inflammatory response [11].

### 2.4. Platelet/Lymphocyte Ratio (PLR)

This parameter is computed as the platelet number over the lymphocyte number. Since platelets are involved in inflammation, the PLR is used as a predictive and prognostic marker in cardiovascular and inflammatory diseases and malignancies [12].

### 2.5. Monocyte/Lymphocyte Ratio (MLR)

It is calculated by the ratio of monocytes to lymphocytes. Since monocytes migrate to the site of inflammation and regulate the immune response, the MLR is an important biomarker in the assessment of systemic inflammation [10].

### 2.6. Statistical Analysis

All statistical analyses were performed using IBM SPSS Statistics software (version 26.0; IBM Corp., Armonk, NY, USA). The Chi-square (χ²) test was used to evaluate the relationships between categorical variables, and Fisher’s exact Chi-square test was preferred for cells with a low number of observations. In the comparison of continuous variables between two groups, Student’s t-test was used for parametric data, and the Mann–Whitney U test was used for non-parametric data based on the assessment of normality. In the comparison of three or more groups, appropriate variance analysis was applied for parametric data, while the Kruskal–Wallis H test was preferred for non-parametric data. The association between the two categorical variables was analyzed using the Pearson Chi-square (χ^2^) test, while the Spearman rank correlation coefficient was employed to assess the relationship between two continuous variables that did not follow a normal distribution.

Stepwise linear regression analysis was performed to determine the factors associated with SIRI, SII, NLR, PLR, and MLR scores. In the first stage, univariate linear regression analyses were performed separately for each dependent variable, and statistically significant variables were determined. In the second stage, only the significant variables were included in the multivariate linear regression model, and the final analysis was performed. Univariate and multivariate logistic regression analyses were performed to determine the factors affecting the risk of preterm delivery. Receiver operating characteristic (ROC) analysis curves were used to determine the diagnostic accuracy of the markers. Statistical significance was set at *p* < 0.05.

### 2.7. Ethics Committee Approval

This study was approved by the Non-Interventional Clinical Research Ethics Committee of the Kafkas University Faculty of Medicine (Approval Date: 10 February 2025, Decision No: 80576354–050–99/616). This study was conducted in accordance with the ethical principles of the Declaration of Helsinki for Human Biomedical Research.

## 3. Results

### 3.1. Comparison of Preterm Delivery Group and Term Group

The number of gravida (*p* = 0.031), parity (*p* < 0.001), and miscarriages (*p* < 0.001) were significantly increased in the control group. The smoking rate (36.9% vs. 13.5%; *p* < 0.001) and socioeconomic status (69.9% vs. 13%; *p* < 0.001) were significantly higher in the preterm delivery group. The proportion of secondary school graduates was significantly higher in the control group (59.7% vs. 80%; *p* < 0.001). The preterm delivery group had significantly lower 1st minute APGAR (*p* < 0.001) and 5th minute APGAR (*p* < 0.001) scores but significantly higher NICU admission rates (35.8% vs. 4%; *p* < 0.001).

In the preterm delivery group, SIRI levels at the time of admission (2.15 (0-16.45) vs. 1.62 (0.01-20.38); p < 0.001) and SII levels in the 1st trimester (898.65 (73.31-5067) vs. 786.25 (17.5-4398.7); p = 0.001) and at the time of admission (917.09 (86.18-3990) vs. 732.17 (15.91-2944); p < 0.001) were significantly higher. NLR levels were significantly higher in the 1st trimester (4.15 (0.47–21.45) vs. 3.58 (0.78–24.85); *p* < 0.001) and at the time of admission (3.83 (0.49–15.06) vs. 3.19 (0.08–12.29); *p* < 0.001); MLR levels were significantly higher at the time of admission (0.307 (0–1) vs. 0.245 (0.06–1.64); *p* < 0.001). The PLR levels were significantly higher in the control group in the 1st trimester (140 (4.5–655.5) vs. 130.19 (28.97–523.3); *p* = 0.019). The detailed data are presented in Table 1.

### 3.2. Comparison of Early Preterm, Middle Preterm, and Late Preterm Delivery Groups

The 1st and 5th minute APGAR scores were significantly lower (*p* < 0.001), while both the rates and duration of NICU admission were significantly higher (*p* < 0.001) in those who delivered in the early preterm period. In the early preterm period, SIRI levels were significantly higher in the 1st trimester (*p* < 0.001) and during admission (*p* < 0.001), SII levels were significantly higher in the 1st trimester (*p* < 0.001) and during admission (*p* < 0.001), and NLR levels were significantly higher in the 1st trimester (*p* < 0.001) and during admission (*p* < 0.001), PLR levels were significantly higher in the 1st trimester (*p* < 0.001) and during admission (*p* < 0.001), and MLR levels were significantly higher in the 1st trimester (*p* < 0.001) and during admission (*p* < 0.001). The detailed data are presented in Table 2.

### 3.3. Factors Affecting the Risk of Preterm Delivery

The SIRI (univariate: OR: 1.057, 95% C.I.: 1.003–1.114, *p* = 0.038; multivariate: OR: 1.345, 95% C.I.: 1.059–1.708, *p* = 0.015) and NLR (univariate: OR: 1.119, 95% C.I.: 1.077–1.163, *p* < 0.001; multivariate: OR: 1. 383, 95% C.I.:1.249–1.532, *p* < 0.001) were both significantly linked to higher odds of preterm birth in univariate and multivariate logistic regression analyses (Table 3).

### 3.4. Receiver Operating Characteristic (ROC) Curve for SIRI, SII, NLR, PLR, and MLR in Predicting Preterm Delivery

**Figure 1:**
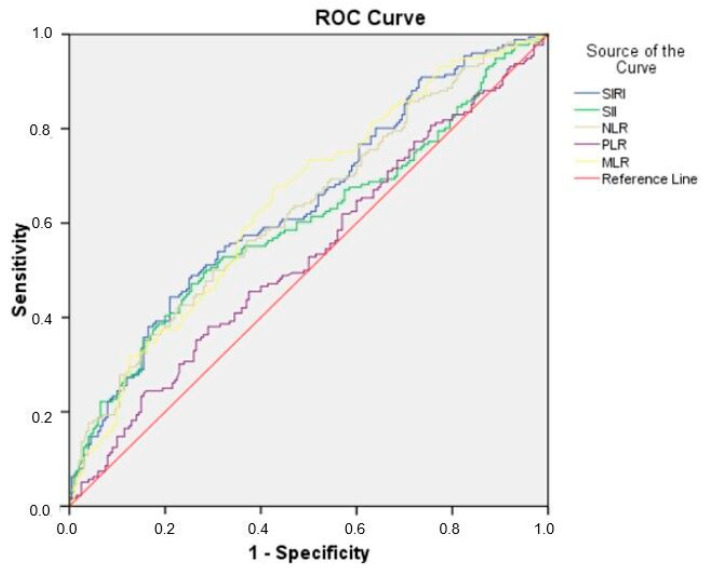
Receiver operating characteristic (ROC) curve for SIRI, SII, NLR, PLR, and MRL in predicting preterm delivery.

The SIRI (AUC: 0.639, cut-off: 1.78, sensitivity: 59.1%, specificity: 59%), NLR (AUC: 0.628, cut-off: 3.44, sensitivity: 58.5%, specificity: 58.5%), and MLR (AUC: 0. 645, cut-off: 0.27, sensitivity: 61.4%, specificity: 61.5%) stood out as the strongest inflammatory markers in predicting preterm labor (*p* < 0.001) (Table 4). These findings support the role of inflammatory response in the pathogenesis of preterm labor and suggest that these markers may be potential biomarkers for clinical use. On the other hand, the diagnostic power of the PLR (AUC: 0.533, cut-off: 126.02, sensitivity: 50%, specificity: 50%) was not significant (*p* = 0.057), whereas the SII (AUC: 0.599, cut-off: 783.48, sensitivity: 56.8%, specificity: 57%) had moderate discriminatory power. ROC analyses of the SIRI, SII, NLR, PLR, and MLR are presented in Figure 1.

## 4. Discussion

In our study, we evaluated the prognostic value of systemic inflammation indices in predicting preterm delivery. SIRI, SII, NLR, and MLR levels were found to be significantly higher in women with preterm delivery. In particular, the SIRI (*p* = 0.015) and NLR (*p* < 0.001) independently increased the risk of preterm delivery in both univariate and multivariate logistic regression analyses. In addition, inflammatory indices were found to be associated with the severity of preterm delivery, with higher levels of these markers in cases of preterm delivery (SIRI: *p* < 0.001, NLR: *p* < 0.001, SII: *p* < 0.001, PLR: *p* < 0.001, MLR: *p* < 0.001). Increases in the SIRI and NLR were significantly associated with decreases in 1st and 5th minute APGAR scores (*p* = 0.029, *p* < 0.001) and increases in NICU admission rates (*p* < 0.001). The PLR was inversely associated with preterm delivery (*p* = 0.002) but not with NICU admission rates (*p* = 0.624). The MLR was also inversely associated with preterm delivery (*p* < 0.001), and increasing MLR levels were associated with a decrease in 5 min APGAR scores (*p* = 0.028) and an increase in NICU admission rates (*p* < 0.001).

In recent years, it has been suggested that defining preterm delivery based solely on gestational age is inadequate and that a new taxonomy that takes into account the underlying etiopathogenetic mechanisms should be adopted instead [13]. This new approach aims to define phenotypes based on infection/inflammation, uteroplacental dysfunction, and immunologic factors. The prominent inflammation markers in our findings, especially the SIRI and NLR, are consistent with this inflammatory phenotype. Moreover, it is also emphasized in the literature that the combined evaluation of cervical length and inflammatory markers is an effective method for predicting spontaneous preterm delivery [14]. On the other hand, placental dysfunction is known to be an important mechanism predisposing to spontaneous preterm delivery; however, it is not yet clear whether infection and inflammation trigger this dysfunction [15]. This possible interaction can be considered as a remarkable and open area of research for future mechanistic studies.

Recent evaluations highlight a potential correlation between the SIRI and the occurrence of preterm delivery. SIRI levels have been found to increase in cases of preterm premature rupture of membranes (PPROM) and may correlate with unfavorable maternal or neonatal outcomes [16]. Some studies have reported that the SIRI is not directly related to the week of birth [1]. However, the SIRI has been reported to be a potential marker for predicting lung maturation and the risk of respiratory distress syndrome (RDS) in preterm infants [17]. High SIRI levels were associated with perinatal complications in pregnant women with Familial Mediterranean Fever (FMF) [18]. In pregnant women with gestational diabetes mellitus (GDM), increased SIRI levels have been shown to be a risk factor for preterm delivery [19]. In our study, an increase in SIRI levels was associated with a significant increase in the preterm delivery rate (*p* = 0.015). Although no difference was observed between the groups in the first trimester, SIRI levels during admission were significantly higher in preterm deliveries (*p* < 0.001). In the preterm group, SIRI levels were higher both in the first trimester and during admission (*p* < 0.001). As the SIRI increased, 1st and 5th minute APGAR scores decreased (*p* < 0.001), while NICU admission rates increased significantly (*p* < 0.001). Our findings suggest that the SIRI may be associated with preterm delivery and neonatal outcomes, supporting this inflammatory index as a potential biomarker.

The role of the SII in predicting preterm delivery and related complications has not been fully clarified. In the literature, it has been reported that SII levels do not differ significantly between preterm delivery and non-preterm delivery, whereas other markers of inflammation are associated with preterm delivery risk [1]. However, in cases of threatened preterm delivery, the SII has been shown to have predictive value and can be used in clinical evaluations [20]. It has also been reported that high SII levels in the first trimester are associated with pre-eclampsia development and should be considered for preterm delivery [21]. In preterm infants, it has been suggested that the SII may be an effective biomarker, like the SIRI, in predicting RDS and lung maturation [17]. In our study, while no significant association was found between SII levels and preterm delivery (*p* = 0.776), SII levels were significantly higher in preterm delivery both in the first trimester and during admission (*p* < 0.001). This difference was more pronounced in the preterm group (*p* < 0.001). In addition, as SII levels increased, 1st minute APGAR score decreased (*p* = 0.004) and NICU admission rates increased (*p* < 0.001). These findings suggest that the SII is not a direct determinant of preterm delivery but may be an important biomarker associated with neonatal prognosis.

The NLR has been reported to predict spontaneous preterm delivery with high sensitivity (90%) and specificity (92.1%) [22]. NLR levels have been shown to be elevated in PPROM cases [23] and have similar predictive power to white blood cell count and CRP in predicting neonatal sepsis and histologic chorioamnionitis [24]. While it has been reported that the NLR is increased in cases of threatened preterm delivery, its predictive power is limited [20]. It has been suggested that it is significantly higher in preterm delivery and may be suitable for clinical use as a low-cost biomarker [25]. In our study, an increase in the NLR was associated with a significant increase in preterm delivery rates (*p* < 0.001). In preterm deliveries, the NLR was significantly higher both in the first trimester and during admission (*p* < 0.001), and this difference was more pronounced in the preterm group (*p* < 0.001). Furthermore, as the NLR increased, the 1st minute APGAR score decreased (*p* = 0.029), and NICU admission rates increased (*p* = 0.009). These findings suggest that the NLR may have clinical value in predicting preterm delivery and neonatal outcomes.

It was reported that the PLR was significantly higher in women with spontaneous preterm delivery [26], and a threshold value of >139 could predict preterm delivery with a sensitivity of 97.5% and specificity of 100% [22]. It has been reported that PLR levels are significantly increased in pregnant women with PPROM and may be an indicator of inflammatory processes [27,28]. It has also been reported that the PLR, like the NLR, can predict the risk of neonatal sepsis [23]. In our study, an increase in the PLR was associated with a significant decrease in preterm delivery rates (*p* = 0.002). In preterm delivery, the PLR was significantly lower in the first trimester (*p* < 0.001). However, no significant association was found between PLR levels and the 1st and 5th minute APGAR scores (*p* = 0.336 and *p* = 0.132, respectively) and NICU admission rates (*p* = 0.624). These results indicate that the PLR could serve as a potential marker for the assessment of preterm delivery risk.

It has been reported that MLR levels are high in women with preterm delivery and may constitute a risk factor, together with other inflammatory indices [1,29]. However, some studies have found no significant differences between preterm and term deliveries and have suggested the evaluation of additional biomarkers to better understand the role of inflammation [30]. It was reported that the MLR increased in cases of PPROM and threatened preterm delivery, but this was not statistically significant [31], and it was associated with GDM and may be an indirect risk factor for preterm delivery [32]. In our study, an increase in the MLR was associated with a significant decrease in preterm delivery rate (*p* < 0.001). However, the MLR levels were significantly higher in preterm deliveries during admission (*p* < 0.001), and this difference was more pronounced in the preterm group (*p* < 0.001). Furthermore, as the MLR increased, 5th minute APGAR scores decreased (*p* = 0.028) and NICU admission rates increased (*p* < 0.001). These findings suggest that the MLR may be a helpful biomarker in monitoring inflammatory processes and predicting neonatal outcomes, rather than being a direct determinant in preterm delivery risk assessment.

A growing number of studies have demonstrated the negative impact of infections on preterm delivery [33,34]. In particular, systemic and intrauterine inflammatory responses triggered by pathogens such as SARS-CoV-2 and Zika virus have been shown to impair fetal oxygenation, disrupt metabolic balance, and adversely affect neurodevelopmental processes, thereby predisposing to preterm delivery [34,35]. Furthermore, SARS-CoV-2 has been suggested to indirectly increase the risk of preterm delivery by disrupting fertility and hormonal balance [36]. Inflammation has been associated not only with the timing of delivery but also with adverse neonatal outcomes, including low umbilical artery pH, reduced APGAR scores, and early neurodevelopmental impairment [2,3,17]. In our study, the significant association between elevated inflammatory indices such as the SIRI and NLR and both low APGAR scores and increased NICU admission rates suggests impaired early neonatal adaptation. These findings support the notion that intrauterine inflammation may exert detrimental effects on fetal well-being and neurodevelopment even from the earliest stages of life.

One of the major strengths of our study is that it provides a comprehensive retrospective analysis evaluating the association between systemic inflammation indices and preterm delivery in a large patient population. As one of the rare studies examining the prognostic value of the SIRI, it makes a unique contribution to the literature by considering this index together with commonly used inflammatory biomarkers such as the NLR, PLR, and MLR. Furthermore, analyzing subgroups according to the severity of preterm delivery, comparing different inflammatory parameters, and excluding maternal diseases that may affect laboratory values increase the reliability of the findings. However, our study had some limitations. The single-center and retrospective design may limit the generalizability of the results. Considering that a single biomarker with high sensitivity and specificity for the prediction of preterm delivery has not yet been identified, well-designed, multicenter investigations with extensive patient cohorts and rigorous methodology are essential to clarify the prognostic utility of inflammatory biomarkers.

## 5. Conclusions

This study demonstrated the important role of systemic inflammatory indices in predicting preterm delivery. Our findings revealed that high levels of the SIRI, SII, NLR, and MLR were strongly associated with preterm delivery risk, whereas the PLR showed an inverse relationship. The prominence of the SIRI and NLR as independent risk factors suggests that these parameters may be used as predictive biomarkers in clinical practice. Furthermore, higher inflammatory indices were associated with lower APGAR scores and increased rates of neonatal intensive care unit admission, emphasizing the impact of systemic inflammation on neonatal outcomes. Although these results provide valuable insights into the inflammatory mechanisms of preterm delivery, larger-scale, multicenter, prospective studies are needed to confirm these findings and investigate their integration into obstetric risk assessments.

## Figures and Tables

**Table 1:** Comparison of preterm group and term group.

	Preterm Group (*n* = 528)	Term Group (*n* = 600)	*p*
Age (years) (Median (Min-Max))	33.5 (19–47)	33.5 (23–53)	0.385
Gravida (Median (Min-Max))	1 (1–6)	2 (1–6)	**0.031**
Parity (Median (Min-Max))	0 (0–4)	1 (0–4)	**<0.001**
Miscarriage (Median (Min-Max))	0 (0–3)	0 (0–3)	**<0.001**
Smoking (*n*, %)	195 (%36.9)	81 (%13.5)	**<0.001**
Employment Status (*n*, %)	195 (%36.9)	210 (%35)	0.500
Education (*n*, %)			
Primary school	144 (%27.3)	69 (%11.5)	**<0.001**
High school	315 (%59.7)	480 (%80)	**<0.001**
College/University	69 (%13.1)	51 (%8.5)	**<0.001**
Yearly income level (*n*, %)			
Low	27 (%5.1)	42 (%7)	**<0.001**
Medium	132 (%25)	480 (%80)	**<0.001**
High	369 (%69.9)	78 (%13)	**<0.001**
Hospitalization Week (Median (Min-Max)	34 (27–36)	39.5 (37–42)	**<0.001**
1st minute APGAR score (Median (Min-Max))	6 (2–7)	8 (7–9)	**<0.001**
5th minute APGAR score (Median (Min-Max))	8 (5–9)	9 (8–10)	**<0.001**
NICU (*n*, %)	189 (%35.8)	24 (%4)	**<0.001**
Duration of NICU Admission (Median (Min-Max))	0 (0–28)	0 (0–3)	**<0.001**
SIRI (Median (Min-Max))			
1st trimester	1.42 (0–16)	1.44 (0.10–26.58)	0.078
Hospitalization Time	2.15 (0–16.45)	1.62 (0.01–20.38)	**<0.001**
SII (Median (Min-Max))			
1st trimester	898.65 (73.31–5067)	786.25 (17.5–4398.7)	**0.001**
Hospitalization Time	917.09 (86.18–3990)	732.17 (15.91–2944)	**<0.001**
NLR (Median (Min-Max))			
1st trimester	4.15 (0.47–21.45)	3.58 (0.78–24.85)	**<0.001**
Hospitalization Time	3.83 (0.49–15.06)	3.19 (0.08–12.29)	**<0.001**
PLR (Median (Min-Max))			
1st trimester	130.19 (28.97–523.3)	140 (4.5–655.5)	**0.019**
Hospitalization Time	126.28 (31.56–417.5)	125.37 (8.26–328.07)	0.057
MLR (Median (Min-Max))			
1st trimester	0.22 (0–1)	0.22 (0.03–2.19)	0.076
Hospitalization Time	0.307 (0–1)	0.245 (0.06–1.64)	**<0.001**

MLR: monocyte/lymphocyte ratio; NLR: neutrophil/lymphocyte ratio; PLR: platelet/lymphocyte ratio; SII: systemic immune-inflammation index; SIRI: Systemic Inflammatory Response Index.

**Table 2:** Comparison of early preterm, middle preterm, and late preterm delivery groups.

	Early Preterm (*n* = 78)	Middle Preterm (*n* = 111)	Late Preterm (*n* = 339)	*p*
Age (years) (Median (Min-Max))	34 (24–44)	34 (23–46)	33 (19–47)	0.667
Gravida (Median (Min-Max))	1 (1–4)	1 (1–5)	1 (1–6)	0.597
Parity (Median (Min-Max))	0 (0–3)	0 (0–3)	0 (0–4)	0.935
Miscarriage (Median (Min-Max))	0 (0–1)	0 (0–3)	0 (0–2)	0.655
Smoking (*n*, %)	21 (%26.9)	36 (%32.4)	138 (%40.7)	**0.041**
Employment Status (*n*, %)	24 (%30.8)	27 (%24.3)	144 (%42.5)	**0.001**
Education (*n*, %)				
Primary school	18 (%23.1)	12 (%10.8)	114 (%33.6)	**<0.001**
High school	51 (%65.4)	75 (%67.6)	189 (%55.8)	**<0.001**
College/University	9 (%11.5)	24 (%21.6)	36 (%10.6)	**<0.001**
Yearly income level (*n*, %)				
Low	0 (%0)	12 (%10.8)	15 (%4.4)	**<0.001**
Medium	15 (%19.2)	9 (%8.1)	108 (%31.9)	**<0.001**
High	63 (%80.8)	90 (%81.1)	216 (%63.7)	**<0.001**
Hospitalization Week (Median (Min-Max))	30 (27–31)	32 (32–33)	35 (34–36)	**<0.001**
1st minute APGAR score (Median (Min-Max))	2 (2–3)	5 (4–6)	6 (6–7)	**<0.001**
5th minute APGAR score (Median (Min-Max))	6 (5–7)	7 (6–8)	8 (8–9)	**<0.001**
NICU (*n*, %)	78 (%100)	90 (%81.1)	21 (%6.2)	**<0.001**
Duration of NICU Admission (Median (Min-Max))	15 (7–28)	7 (0–7)	0 (0–10)	**<0.001**
SIRI (Median (Min-Max))				
1st trimester	5.72 (4.07–16)	2.76 (1.87–4.39)	0.94 (0–2.3)	**<0.001**
Hospitalization Time	6.89 (5.24–16.45)	3.5 (3.01–4.93)	1.45 (0–2.96)	**<0.001**
SII (Median (Min-Max))				
1st trimester	2035 (808–4089)	1212 (640–3456)	625.9 (73.3–5067)	**<0.001**
Hospitalization Time	1930 (855–3262)	1225 (680–3217)	641 (86–3990)	**<0.001**
NLR (Median (Min-Max))				
1st trimester	10.2 (4.57–21.45)	5.94 (3.02–10.85)	3.37 (0.479–20.6)	**<0.001**
Hospitalization Time	9.1 (4.37–15.06)	5.20 (2.92–8.67)	3.11 (0.49–15)	**<0.001**
PLR (Median (Min-Max))				
1st trimester	171.8 (63.6–426)	140 (61.53–320)	121.8 (28.9–523.3)	**<0.001**
Hospitalization Time	158 (65.7–332.85)	134.46 (64.16–292)	118.5 (31.5–417.5)	**<0.001**
MLR (Median (Min-Max))				
1st trimester	0.54 (0.29–1)	0.32 (0.17–0.69)	0.16 (0–0.38)	**<0.001**
Hospitalization Time	0.59 (0.34–1)	0.40 (0.26–0.75)	0.25 (0–0.46)	**<0.001**

MLR: monocyte/lymphocyte ratio; NLR: neutrophil/lymphocyte ratio; PLR: platelet/lymphocyte ratio; SII: systemic immune; inflammation index; SIRI: systemic inflammatory response index.

**Table 3:** Factors affecting the risk of preterm delivery.

	Univariate Analysis				Multivariate Analysis	
	aOR	95% C.I	*p*	aOR	95% C.I	*p*
Age (years) (Median (Min-Max))	1.009	0.989–1.030	0.388	1.015	0.990–1.039	0.241
Gravida (Median (Min-Max))	0.941	0.843–1.050	0.276	1.566	1.043–2.351	**0.031**
Parity (Median (Min-Max))	0.795	0.700–0.903	**<0.001**	0.553	0.365–0.837	**0.005**
Miscarriage (Median (Min-Max))	1.586	1.166–2.158	**0.003**	1.439	0.858–2.415	0.168
Smoking (*n*, %)	0.267	0.199–0.357	**<0.001**	0.246	0.177–0.342	**<0.001**
Employment Status (*n*, %)	1.088	0.852–1.388	0.500	0.951	0.714–1.268	0.733
1st trimester (Median (Min-Max))						
SIRI	1.057	1.003–1.114	**0.038**	1.345	1.059–1.708	**0.015**
SII	1.0	1.000–1.001	**<0.001**	1.0	0.999–1.001	0.776
NLR	1.119	1.077–1.163	**<0.001**	1.383	1.249–1.532	**<0.001**
PLR	0.998	0.997–1.0	0.066	0.993	0.989–0.997	**0.002**
MLR	0.586	0.309–1.112	0.102	0.001	0.000–0.008	**<0.001**

MLR: monocyte/lymphocyte ratio; NLR: neutrophil/lymphocyte ratio; PLR: platelet/lymphocyte ratio; SII: systemic immune-inflammation index; SIRI: systemic inflammatory response index.

**Table 4:** Receiver operating characteristic (ROC) analysis for preterm delivery.

Variables	AUC	S.E.	*p*	OR (95% CI)	Cutt-Off	Sensitivity	Specificity
SIRI	0.639	0.016	**<0.001**	0.607–0.671	1.78	59.1%	59%
SII	0.599	0.017	**<0.001**	0.566–0.633	783.48	56.8%	57%
NLR	0.628	0.017	**<0.001**	0.596–0.660	3.44	58.5%	58.5%
PLR	0.533	0.017	0.057	0.499–0.567	126.02	50%	50%
MLR	0.645	0.016	**<0.001**	0.613–0.677	0.27	61.4%	61.5%

AUC: area under the curve; MLR: monocyte/lymphocyte ratio; NLR: neutrophil/lymphocyte ratio; PLR: platelet/lymphocyte ratio; S.E.: standard error; SII: systemic immune-inflammation index; SIRI: systemic inflammatory response index.

## Data Availability

All data related to this study have been included in the article.

## Abbreviations

CRP	C-Reactive Protein
FMF	Familial Mediterranean Fever
GDM	Gestational Diabetes Mellitus
IL-6	Interleukin-6
MLR	Monocyte/Lymphocyte Ratio
MMP-9	Matrix Metalloproteinase-9
NICU	Neonatal Intensive Care Unit
NLR	Neutrophil/Lymphocyte Ratio
PLR	Platelet/Lymphocyte Ratio
PPROM	Preterm Premature Rupture of Membranes
RDS	Respiratory Distress Syndrome
ROC	Receiver Operating Characteristic
SII	Systemic Immune-Inflammation Index
SIRI	Systemic Inflammatory Response Index
TNF-α	Tumor Necrosis Factor-alpha
